# Uterine artery Doppler indices throughout gestation in women with and without previous Cesarean deliveries: a prospective longitudinal case–control study

**DOI:** 10.1038/s41598-022-25232-z

**Published:** 2022-12-03

**Authors:** Piengbulan Yapan, Suphaphon Tachawatcharapunya, Supitchaya Surasereewong, Piyatida Thongkloung, Julaporn Pooliam, Liona C. Poon, Tuangsit Wataganara

**Affiliations:** 1 Division of Maternal-Fetal-Medicine, Department of Obstetrics and Gynecology, Faculty of Medicine Siriraj Hospital, Mahidol University, 2 Prannok Road, Bangkoknoi, Bangkok 10700 Thailand; 2 Division of Clinical Epidemiology, Faculty of Medicine Siriraj Hospital, Mahidol University, Bangkoknoi, Thailand; 3 Department of Obstetrics and Gynaecology, Prince of Wales Hospital, The Chinese University of Hong Kong, Shatin, Hong Kong SAR China

**Keywords:** Biomarkers, Risk factors

## Abstract

To determine whether a history of previous Cesarean delivery (CD) impacts uterine artery (UtA) Doppler indices throughout pregnancy. Women with and without CD (NCD) were prospectively enrolled for sequential assessments of the UtA mean/median pulsatility index (UtA-PI), resistance index (UtA-RI), and systolic/diastolic ratio (UtA-S/D) at 11–13 + 6, 14–19 + 6, 30–34 + 6, and 35–37 + 6 weeks’ gestation. Data from 269/269, 246/257, 237/254, and 219/242 CD/NCD participants from each gestational period were available for analysis. Multiples of the median (MoMs) of UtA Doppler indices showed biphasic temporal (Δ) pattern; with an initial dropping until the second trimester, then a subsequent elevation until late in pregnancy (*p* < 0.05). The measurements and Δs of the UtA indices between CD and NCD were not different (*p* > 0.05). Mixed-effects modelling ruled out effects from nulliparity (n = 0 and 167 for CD and NCD, respectively) (*p* > 0.05). History of CD neither influenced the measurements nor the temporal changes of the UtA Doppler indices throughout pregnancy. The biphasic Δs of UtA Doppler indices added to the longitudinal data pool, and may aid in future development of a more personalized prediction using sequential/contingent methodologies, which may reduce the false results from the current cross-sectional screening.

## Introduction

Cesarean delivery (CD) has been linked with an impaired fertility in subsequent pregnancies, partly due to miscarriages and placental related complications^1^. The steep rise in the CD rate is attributed to changes in the maternal characteristics; including parity, age, and body mass index (BMI)^2^. These variables are reciprocally well-established predictors for the placental related complications, i.e. preeclampsia (PE), which is a significant contributor to maternal and fetal morbidities^3^. Because the uterine artery Doppler pulsatility index (UtA-PI) may be elevated prior to the clinical manifestation of PE, integrating the first-trimester (11–13 weeks of gestation) mean UtA-PI multiples of the median (MoMs) with maternal characteristics using Bayes’s theorem-based multimodal prediction modeling developed by the Fetal Medicine Foundation (FMF; London, United Kingdom) has improved the predictive value for preterm PE to 76.7% (with a risk cut-off of 1 in 100 cutoff, 10.5% screen-positive rate (SPR), and 9.2% false positive rate (FNR)^4,5^. The FMF’s first-trimester prediction modeling aims to identify women who would benefit from early administration of aspirin to mitigate obstetric morbidities associated with preterm PE and FGR^6,7^. It has been validated globally (except in Africa), and can be conveniently integrated with the routine first-trimester combined test for Down syndrome. The detection rate (DR) of term PE (> / = 37 weeks’ gestation) by FMF’s algorithm was only 43.1% at the same SPR and FPR^5^. There are still needs to reduce FPR for preterm PE prediction to minimize unnecessary administration of aspirin, and to reduce the false negative rate (FNR) for term PE prediction for a more effective mitigation of PE-related morbidities^8^. Cross-sectional screening in the second, early-third, or late-third trimester of pregnancy (19–24, 30–34, and 35–37 weeks of gestation, respectively) however, performed even poorer than the first trimester, mainly due to varying FPRs and DRs^9–11^.

Sequential and contingent approaches consider temporal changes (Δs) of UtA-PI and serum biomarkers subsequently screened following the first trimester of pregnancy, which may lower the FPR and FNR for prediction of preterm PE by over 25% and 50%, respectively^12^. Alternatively, serum biomarkers may be excluded, and only MAP, BMI, and UtA-PI (measured opportunistically during the routine second-trimester scanning without additional cost) are considered for in the sequential and contingent approaches^10^. As a stand-alone marker, the DR of preterm PE with first-trimester UtA-PI measurement was 30.8–47.8%, depending on the cutoffs^13,14^. Previous cross-sectional and semi-longitudinal studies consistently showed a progressive decrease of the mean UtA-PI MoMs until late stages of pregnancy^13,15,16^. Persistently high or steeper decrease in the mean UtA-PI MoMs between the first and second trimester of pregnancy may improve the specificity of prediction in women with the highest risk after combined first-trimester screening^13,15,17–20^. Longitudinal methodology to study Δ mean UtA-PI MoMs throughout gestation would provide the opportunity to resolve much of the concomitant ambiguity, and would enhance the development of personalized sequential prediction model.

Parity, but not route of delivery in previous pregnancies, is an established confounder that needs to be adjusted for the first-trimester mean UtA-PI MoMs as per the FMF’s algorithm^21,22^. On the other hand, UtA Doppler measurements in the second (18–22 and 22–24 weeks’ gestation) and third (26–32 weeks’ gestation) trimester of pregnancy demonstrated a trend toward increased uterine vascular resistance in women with previous CD compared to those without previous CD (NCD)^23–25^. In theory, laceration or kinking of the UtAs from low-transverse uterine incision and suture repair at the level of uterine isthmus where the left and right UtAs are clinically interrogated as per a standardized technique may explain the consequent changes of UtA hemodynamics^17^. Pre-existing aberrations of UtA hemodynamics may have led to CD in previous pregnancies. To our knowledge, there have been no published evidence if the type or the number of CD might affect UtA Doppler measurements. Since CD has become epidemic, our study looked to determine the personalized impacts of CD on the Δ mean UtA-PI MoMs using longitudinal methodology.

## Methods

### Study design and population

This was a prospective observational case–control study. Participants were Thai women who attended antenatal care at Department of Obstetrics and Gynecology, Faculty of Medicine Siriraj Hospital, Bangkok, Thailand from September 2018 to January 2020, with the last delivery in August 2020. The inclusion criteria were singleton pregnancy, gestational age of 11–13 + 6 weeks confirmed by fetal crown-rump length (CRL) measurement, over the age of 18 years, and with written consent for the study. Exclusion criteria were major fetal anomalies detected from first-trimester scanning. The CD and NCD participants were further classified as nulliparous and multiparous (previous vaginal delivery). Characteristics of all eligible participants were collected. Pregnancy outcomes were prospectively collected using the research’s checklist electronic form; which included gestational age at delivery, mode of delivery, birthweight, preterm delivery, gestational diabetes mellitus (DM), gestational hypertension, PE, placental abruption, oligohydramnios, stillbirth, intrauterine fetal death, small for gestational age infant (birthweight less than 10th percentile according to World Health Organization (WHO) fetal growth chart)^26^.

Transabdominal ultrasound examinations were performed by FMF-certified examiners using Voluson E6, Voluson E8, or Voluson E10 ultrasound machines with 2–5 MHz transducer (GE Healthcare, Vienna, Austria). All study participants provided the informed consent for 4 consecutive ultrasound examinations at gestational age intervals of 11–13 + 6, 19–24 + 6, 30–34 + 6, and 35–37 + 6 weeks^7,9–11^. Placental location was recorded in each scan. Mean UtA-PI, mean resistance index (UtA-RI), and median systolic/diastolic (UtA-S/D) ratio were recorded with automatic Doppler waveform measurement as per the established protocols as follows.

#### *First trimester scan: 11–13 *+ *6 weeks of gestation*

Fetal CRL was measured according to the standard protocol^27^. Right and left UtAs were identified with color Doppler at the level of internal cervical os in sagittal view. Pulse-wave Doppler, with a sample gate of 2 mm and angle of insonation below 30 degrees, was applied to acquire 3 consecutive waveforms with peak systolic velocity (UtA-PSV) above 60 cm/s before the measurement was made^28^. The pre-diastolic notching was not considered in this study due to its subjective nature of assessment, which may be inaccurate particularly under pressure of the examination time^29^.

#### *Second trimester scan: 19–24 *+ *6 weeks of gestation, third trimester scan: 30–34 *+ *6 weeks of gestation, and near delivery scan: 35–37 *+ *6 weeks of gestation*

Fetal biparietal diameter (BPD), head circumference (HC), abdominal circumference (AC), femur length (FL) and estimated fetal weight (EFW) were measured from second trimester scan onward. Amniotic fluid index (AFI) and pulse-wave Doppler studies of the umbilical and middle cerebral arteries were additionally measured in third trimester and near delivery scans as part of research protocol. With the transducer placed at inguinal canal, right and left uterine arteries were identified with color Doppler at the point where they crossed over the iliac vessels. Pulse-wave Doppler, with a sample gate of 2 mm taken approximately 1 cm distal to the crossing point and angle of insonation below 30 degrees, was applied to acquire 3 consecutive waveforms with UtA-PSV above 60 cm/s before the measurement was made^30^.

The study protocol was approved by the Siriraj Institutional Review Board (SIRB), Faculty of Medicine Siriraj Hospital, Mahidol University, Bangkok, Thailand (COA no. Si 588/2018). All experiments were performed in accordance with relevant guidelines and regulations. The 11–13 + 6 weeks’ data were nested in Asia-wide validation of the first-trimester screening for preterm PE by the FMF-based method and non-interventional phase of First-trimester Screening and preventiOn of pREeClAmpSia Trial (FORECAST)^31^. All the participants received aspirin prophylaxis as per recommendation of the National Institute for Care and Health Excellence (NICE)^32^. The FORECAST trial was approved by the Joint Chinese University of Hong Kong New Territories East Cluster Clinical Research Ethics Committee (CREC Ref. No. 2016.152) in Hong Kong, and is registered with ClinicalTrials.gov (Identifier: NCT03554681).

### Sample size calculation and statistical analysis

Recent systematic review suggested that UtA-PIs are more consistent and may be more predictive if measured in the second trimester of pregnancy^29^. Therefore a previously published case–control study conducted between 18 and 22 weeks’ gestation that reported the mean ± standard deviation (SD) of UtA-PI in CD (n = 200) and NCD (n = 200) of 1.25 ± 0.4 and 1.16 ± 0.34, respectively (*p* = 0.02) was chosen for calculation of sample size^23^. To be conservative, type I error was fixed at a maximum value of 5% and an 80% power, which gave an estimate of 269 participants required for the CD and NCD groups. We used two-sided *p* value to test the hypothesis, and set significance at *p* < 0.05. Statistical analyses were carried out using PASW Statistics (SPSS) 18.0 (SPSS Inc., Chicago, IL., USA). Mean (± SD) represented an average PIs and RIs measured from the left and the right UtAs. Median (minimum–maximum) represented an average S/D ratio. To be conservative, statistical analyses were performed in the scale of logarithmic MoM to allow for Gaussian distribution of the measurements. A linear mixed-effects model for normally distributed continuous data repeated measures was used to analyze the difference of UtA indices MoMs between CD and NCD groups and changes overtime within group. One-way ANOVA and Kruskal Wallis test with post-hoc test by Bonferroni was used to analyze the difference of UtA indices MoMs between nulliparity (Nullip), previous vaginal delivery (Prev Vg), and CD groups. Linear regression analysis was use to find the best fit models between mean UtA-PI MoMs and pre-pregnancy body mass index (BMI), chronic hypertension, overt DM, renal, and cardiac diseases.

## Results

**Figure 1:**
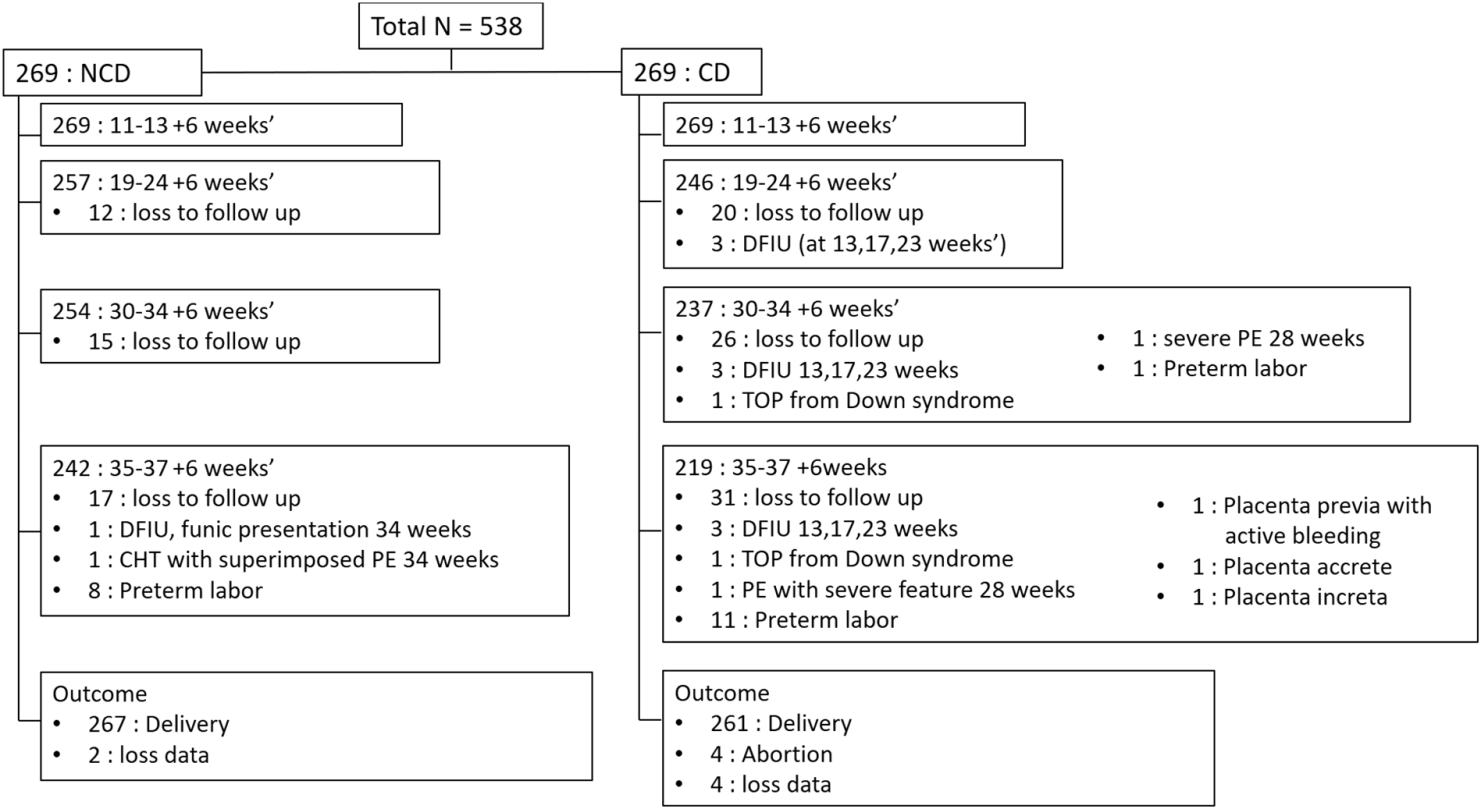
Enrollment scheme. *NCD* no Cesarean delivery, *CD* Cesarean delivery, *DFIU* dead fetus in utero, *CHT* chronic hypertension, *PE* preeclampsia, *TOP* termination of pregnancy.

Maternal characteristics of 538 participants in CD (n = 269) and NCD (n = 269) groups are summarized in Table 1. The age, parity, and pre-pregnancy BMI of women in CD group were higher than those in NCD groups. (*p* < 0.01) The unprecedented outbreak of severe acute respiratory syndrome coronavirus-2 (SARS-CoV-2)-associated infection (COVID-19) affected the adherence of the participants. Data from 269/269, 246/257, 237/254, and 219/242 CD/NCD participants from each gestational age intervals were available for analysis (Fig. 1). Of noted, among CD subjects, 261/269 (97%) had 1 previous CD, whereas 8/269 (3%) had > / = 2 previous CDs.

**Table 1:** Maternal demographic data reported as number and percentage (%), and mean ± standard deviation.

Variables	CD (n = 269)	NCD (n = 269)	*p*-value
Maternal age (years)	32.57 (4.65)	29.69 (5.49)	< 0.001*
18–34	171 (63.6%)	212 (78.8%)	< 0.001*
≥ 35	98 (36.4%)	57 (21.2%)	
Parity			
Nullip	0 (0%)	167 (62.1%)	< 0.001*
Multip	269 (100%)	102 (37.9%)	
Pre-pregnancy BMI (kg/m^2^)	24.89 ± 4.93	23.20 ± 5.06	< 0.001*
< 18.5	14 (5.2%)	42 (15.6%)	< 0.001*
18.5–22.9	96 (35.7%)	113 (42.0%)	
23–24.9	47 (17.5%)	40 (14.9%)	
≥ 25	112 (41.6%)	74 (27.5%)	
Gestational age at enrollment	12.14 ± 0.62	12.16 ± 0.67	0.737
Medical conditions			
Chronic hypertension	9 (3.3%)	12 (4.5%)	0.657
Overt DM	3 (1.1%)	1 (0.4%)	0.373
Renal disease	1 (0.4%)	3 (1.1%)	0.624
Cardiac disease	3 (1.1%)	3 (1.1%)	1.000
SLE	1 (0.4%)	3 (1.1%)	0.624
APS	0 (0%)	0 (0%)	–
Family history of preeclampsia	0 (0%)	0 (0%)	–
Smoking status	0 (0%)	0 (0%)	–
Use of antihypertensive medications	8 (3.0%)	12 (4.5%)	0.495
Use of aspirin	22 (8.2%)	15 (5.6%)	0.307
Placental location			
Anterior	129 (48.0%)	112 (41.6%)	0.325
Posterior	116 (43.1%)	122 (45.4%)	
Lateral	20 (7.4%)	30 (11.2%)	
Fundus	4 (1.5%)	5 (1.9%)	

*CD* previous Cesarean delivery, *NCD* no previous Cesarean delivery, *Nullip* nulliparity, *Multip* multiparity, *BMI* body mass index, *kg/m*^*2*^ kilograms per square meters, *DM* diabetes mellitus, *SLE* systemic lupus erythematosus, *APS* antiphospholipid syndrome.

*p value < 0.05 is considered statistically significant.

Significant values are in [bold].

Longitudinal alterations of MoMs of the mean UtA-PI, mean UtA-RI, and median UtA-S/D in both groups showed a significant biphasic pattern; an initial dropping between the first and the second trimester of pregnancy, followed by a continuous elevation from the second trimester, early and late third trimester of pregnancy (*p* < 0.05). The Δs of mean UtA-PI MoMs were more robust (*p* < 0.01) compared to those of the UtA-RI and UtA-S/D (*p* < 0.05) (Table 2). There was no significant difference in the measurements and the Δs of the mean UtA-PI, mean UtA-RI, and median UtA-S/D MoMs between CD and NCD (*p* > 0.05) (Table 2).

**Table 2:** Longitudinal alterations of multiples of the median (MoMs) of mean uterine artery pulsatility index (UtA-PI), mean resistance index (UtA-RIs), and median systolic/diastolic ratio (UtA-S/D) throughout gestation reported as mean ± standard deviation (SD) and median (minimum–maximum).

	UtA-PI MoMs# (mean ± SD)			UtA-RI MoMs^#^ (mean ± SD)			UtA-S/D MoMs^#^ (median (minimum–maximum))		
	CD	NCD	*p*-value	CD	NCD	*p*-value	CD	NCD	*p*-value
11–13 + 6 (‘First’)	1.11 ± 0.34	1.08 ± 0.33	0.281	1.05 ± 0.14	1.04 ± 0.13	0.161	1.13 (0.47–22.68)	1.06 (0.42–10.46)	0.057
19–24 + 6 (‘Second’)	0.82 ± 0.31	0.81 ± 0.27	0.646	0.93 ± 0.15	0.92 ± 0.15	0.489	0.81 (0.53–2.65)	0.79 (0.39–2.01)	0.433
30–34 + 6 (‘Third’)	0.99 ± 0.32	0.94 ± 0.26	0.051	0.99 ± 0.17	0.97 ± 0.16	0.120	0.95 (0.73–2.48)	0.93 (0.66–2.10)	0.083
35–37 + 6 (‘Near-Delivery’)	1.20 ± 0.35	1.17 ± 0.35	0.278	1.07 ± 0.18	1.05 ± 0.18	0.218	1.06 (0.78–3.83)	1.05 (0.79–3.31)	0.346
*p*-value	< 0.01*	< 0.01*	N/A	< 0.05*	< 0.05*	N/A	< 0.05*	< 0.05*	N/A
**Δ**									
First–Second	0.284 ± 0.364	0.262 ± 0.284	0.478	0.121 ± 0.148	0.112 ± 0.142	0.594	0.296 (− 1.357, 21.912)	0.213 (− 0.969, 9.265)	0.060
First–Third	0.120 ± 0.417	0.139 ± 0.336	0.584	0.060 ± 0.194	0.066 ± 0.171	0.725	0.144 (− 1.504, 21.845)	0.095 (− 0.938, 9.055)	0.282
First-Near Delivery	− 0.093 ± 0.423	− 0.093 ± 0.424	0.986	− 0.021 ± 0.192	− 0.017 ± 0.194	0.782	0.020 (− 2.283, 21.821)	− 0.010 (− 2.194, 9.457)	0.238
Second–third	− 0.169 ± 0.369	− 0.127 ± 0.268	0.224	− 0.063 ± 0.162	− 0.047 ± 0.161	0.307	− 0.144 (− 1.714, 0.648)	− 0.125 (− 0.823, 0.871)	0.275
Second-Near Delivery	− 0.384 ± 0.370	− 0.361 ± 0.367	0.521	− 0.146 ± 0.169	− 0.130 ± 0.190	0.314	− 0.260 (− 2.855, 0.458)	− 0.240 (− 2.243, 0.849)	0.422
Third-Near Delivery	− 0.223 ± 0.363	− 0.226 ± 0.344	0.992	− 0.082 ± 0.193	− 0.080 ± 0.186	0.878	− 0.107 (− 2.874, 1.458)	− 0.109 (− 2.146, 1.129)	0.814
	< 0.01*	< 0.01*	N/A	< 0.05*	< 0.05*	N/A	< 0.05*	< 0.05*	N/A

*GA* gestational age, *CD* previous Cesarean delivery, *NCD* no previous Cesarean delivery, *N/A* not available, ***Δ*** difference.

*p value < 0.05 is considered statistically significant.

^#^Linear mixed model for repeated measures was used to analyze the difference between groups and changes overtime within group.

Significant values are in [bold].

Mixed-effects model shows that nulliparity (n = 0 and 167 for CD and NCD, respectively) did not affect the comparison of the mean UtA-PI, mean UtA-RI, and median UtA-S/D MoMs and their Δs between CD and NCD (*p* > 0.05) (Table 3). Overall, the pregnancy outcomes between CD and NCD participants (n = 265 and 267, respectively) were not different, except for the gestational age at delivery and incidence of small-for-gestational age infants (Table 4).

**Table 3:** Longitudinal alterations of multiples of the median (MoMs) of uterine artery pulsatility index (UtA-PI), resistance index (UtA-RIs), and systolic/diastolic ratio (UtA-S/D ratio) throughout gestation according to mode of delivery in previous pregnancies reported as mean (95% confidence interval).

GA intervals (weeks)	UtA-PI MoMs^#^ (mean (95% CI))				UtA-RI MoMs^#^ (mean (95% CI))				UtA-S/D ratio MoMs^#^ (mean (95% CI))			
	Nullip	Prev Vg	CD	*p*-value	Nullip	Prev Vg	CD	*p*-value	Nullip	Prev Vg	CD	*p*-value
11–13 + 6	1.118 (1.068–1.168)	1.020 (0.956–1.084)	1.112 (1.072–1.151)	0.053	1.044 (1.024–1.065)	1.022 (0.996–1.048)	1.052 (1.036–1.068)	0.161	1.123 (1.053–1.199)	1.025 (0.944–1.113)	1.169 (1.110–1.230)	0.054
19–24 + 6	0.842 (0.797–0.887)	0.768 (0.711–0.825)	0.826 (0.789–0.862)	0.160	0.930 (0.906–0.953)	0.911 (0.881–0.940)	0.932 (0.913–0.950)	0.725	0.848 (0.814–0.884)	0.803 (0.762–0.846)	0.841 (0.814–0.869)	0.310
30–34 + 6	0.959 (0.914–1.004)	0.915 (0.858–0.971)	0.994 (0.956–1.032)	0.081	0.976 (0.950–1.001)	0.960 (0.928–0.992)	0.993 (0.971–1.015)	0.375	0.967 (0.937–0.998)	0.944 (0.907–0.983)	0.998 (0.971–1.025)	0.076
35–37 + 6	1.178 (1.121–1.234)	1.163 (1.092–1.234)	1.209 (1.159–1.260)	0.875	1.045 (1.017–1.074)	1.061 (1.025–1.098)	1.073 (1.047–1.099)	1.000	1.078 (1.041–1.115)	1.105 (1.058–1.153)	1.109 (1.075–1.144)	0.668
*p*-value	< 0.01*	< 0.01*	< 0.01*	N/A	< 0.05*	< 0.05*	< 0.05*	N/A	< 0.05*	< 0.05*	< 0.05*	N/A

*GA* gestational age, *Nullip* nulliparity, *Prev Vg* previous vaginal delivery, *CD* previous Cesarean delivery, *CI* confidence interval, *N/A* not available.

*p-value < 0.05 is considered statistically significant for all gestational age intervals within each mode of delivery in previous pregnancies.

^#^Mixed effect model for repeated measures was used to analyze the difference between groups and changes overtime within group.

Significant values are in [bold].

**Table 4:** Pregnancy outcomes reported as number and percentage (%) and mean ± standard deviation (SD)between no previous Cesarean delivery (NCD) and previous Cesarean delivery (CD) groups reported as number and percentage or mean ± standard deviation or median (minimum–maximum).

Pregnancy outcomes	CD (n = 265)	NCD (n = 267)	*p*-value
Gestational age at delivery (weeks of gestation)	37.43 ± 2.67	38.40 ± 1.29	< 0.001*
Mean birthweight (grams)	3097.97 ± 449.67	3058.15 ± 428.78	0.298
Delivery			
Normal delivery	1 (0.4%)	166 (62.2%)	< 0.001*
Vacuum extraction	1 (0.4%)	8 (3%)	
Cesarean delivery	259 (97.7%)	93 (34.8%)	
Abortion	4 (1.5%)	0 (0%)	
Preterm delivery	24 (9.1%)	14 (5.2%)	0.095
Spontaneous preterm delivery (< 37 weeks’)	18 (75.0%)	13 (92.9%)	
Indicated preterm delivery (> / = 37 weeks’X	6 (25.0%)	1 (7.1%)	
Preeclampsia	9 (3.4%)	9 (3.4%)	1.000
Gestational hypertension	5 (1.9%)	4 (1.5%)	0.751
Gestational diabetes mellitus	53 (20.0%)	33 (12.3%)	0.093
GDMA1	44 (16.7%)	28 (10.5%)	
GDMA2	6 (2.3%)	4 (1.5%)	
Overt DM	3 (1.1%)	1 (0.4%)	
Intrauterine fetal demise	3 (11.3%)	1 (0.4%)	0.371
Small for gestational age			
(Birthweight < 10th percentile)	26 (9.8%)	23 (8.1%)	0.98
Oligohydramnios	0 (0.0%)	4 (1.5%)	0.124
Placental abruption	0 (0.0%)	0 (0.0%)	N/A

*GDMA1* gestational diabetes mellitus type1, *GDMA2* gestational diabetes mellitus type2, *Overt DM* overt diabetes mellitus.

*p value < 0.05 is considered statistically significant.

Significant values are in [bold].

## Discussion

### Principal findings

This study showed that route of delivery in previous pregnancies did not independently influence either the measurements or the Δs MoMs of the mean UtA-PI, mean UtA-RI, and median UtA-S/D throughout pregnancy. It may be safe to say that the uterus is so resilient to blood supply, but this finding cannot be over-extrapolated to lack of impact of CD to reproductive health because the number of previous CDs was not controlled in this study^33^. For prediction with current FMF’s algorithm, there is no need to adjust the mean UtA-PI MoMs for the CD women for cross-sectional screening in either the first, second, early third, and late third trimester of pregnancy. Maternal history of previous CD does not concern the future development of personalized sequential prediction model for preterm PE^34^.

### Secondary findings

Counterintuitively, the Δs of the mean UtA-PI, mean UtA-RI, and median UtA-S/D MoMs in this cohort were concordant for biphasic pattern, which was contradicting with previous studies^13,15^. It was shown in our previous publication that the median UtA-PI MoM value was not significantly deviated from that obtained from European population^35^. Therefore, the discrepancy was likely a consequence of different study methodologies, and this biphasic Δs pattern is more suitable for future development of personalized prediction of PE with sequential/contingent approaches based on UtA-PI measurements. The Δs of the mean UtA-PI MoMs between the first and the second trimester of pregnancy (steep reduction) seem to be more robust than the other intervals (gradual elevation) from the second, early third, and late third trimester of pregnancy (Table 3), but our study was not powered to validate this observation. The progressive reduction Δs pattern of the mean UtA-PI MoMs until late stages of pregnancy in previous publications was supported by data from pre-clinical studies of the uterine arterial system remodeling from the paracrine effects of trophoblastic invasion were mostly done in primates^36^. The finding of biphasic Δs pattern of the mean UtA-PI MoMs suggests that the physiologic changes may well be induced by a more complex endocrine milieu, i.e. calcium ion signaling, endothelial cell–cell contacts, that undergo varying degree of transformation at different stages of pregnancy^37,38^. Therefore, it is conceivable that PE prediction model based on UtA-PI may be increasingly inaccurate in the third trimester of pregnancy from the impacts of gestational changes, particularly those involving the endothelial responsiveness. The UtA-based prediction model of FGR, on the other hand, was able to consider the longitudinal Δs log10UtA-PI between second and early third trimester (20 and 28–34 weeks’ gestation, respectively) because this disease entity does not concern the endothelial dysfunction as in PE^39^. However, currently available information do not support the cost-effectiveness of UtA Doppler assessment in either the early or late third trimester of pregnancy^40,41^. Serum biomarkers, i.e. pregnancy-associated plasma protein A (PAPP-A), placental growth factors (PlGF), soluble fms-like tyrosine kinase-1 (sFlt-1), and soluble endoglin, are more representative for the extent of apoptosis of the placenta, as well as endothelial function or dysfunction, that lead to the imminent manifestation PE-related phenotypes^42,43^.

### Limitations of the study

Our prospective study was not powered to compare the robustness of Δs of the mean UtA-PI MoMs among gestational age intervals.

### Conclusions and future trajectories

Our results concluded that previous CD did not independently influence either the measurements or the alterations of the uterine artery Doppler indices in subsequent pregnancy. Secondary findings add to the pool of longitudinal data for development of UtA-PI-based sequential screening. Recent evidence showed a substantial improvement in the DR for preterm and term PE by sequential approach of maternal characteristics and serum biomarker, but not UtA-PI, data longitudinally collected after 20 and 32 weeks’ gestation, respectively^34^. Prediction features including the screening variables, the approach, and the algorithm may need to be tailored accordingly with the immediate availability of standardized UtA Doppler assessment and automated quantitation of serum biomarkers in individual practice. Preliminary data suggest the feasibility to train the machine to recognize multiple layers of information (i.e. Δs pattern) and its metadata for making an automated decision to the prediction features that will yield the highest predictive performance for preterm and term PE^44,45^.

## Data Availability

Data available on request from the corresponding author (T.W.).
